# Self-care practice and its associated factors among diabetic patients attending public hospitals in Gurage zone southwest, Ethiopia

**DOI:** 10.1371/journal.pone.0271680

**Published:** 2022-09-26

**Authors:** Mamo Solomon Emire, Bitew Tefera Zewudie, Tadesse Tsehay Tarekegn, Fisha Alebel GebreEyesus, Baye Tsegaye Amlak, Shegaw Tesfa Mengist, Tamene Fetene Terefe, Agerie Aynalem Mewahegn

**Affiliations:** Department of Nursing, Wolkite University College of Medicine and Health Science, Wolkite, Ethiopia; University of Turin, ITALY

## Abstract

**Background:**

Diabetes is a chronic disease that requires lifelong medical treatment and lifestyle modifications. Even though patients often neglect their own needs, self-care is an important factor in preventing and delaying complications related to diabetes. There are limited studies about self-care practice, and most of the studies conducted in Ethiopia focused on some parts of the recommended self-care practice. Therefore, this study aimed to assess the self-care practice and associated factors among diabetic patients in Gurage zone, south Ethiopia.

**Methods:**

An institution-based cross-sectional study was conducted from February 6 to March 29, 2021. A systematic sampling method was employed to select 420 study participants. The data were collected using a pretested interviewer-administered questionnaire. All variables with P < 0.25 in the bi-variable logistic regression analysis were entered into multivariable logistic regression analysis. The statistical significance was declared at a p-value < 0.05.

**Results:**

A total of 384 diabetes patients participated with a response rate of 91.4%. This study showed that more than half (60.4%) of the study participants had poor self-care practices. Being female (AOR: 2.40; 95% CI:1.31–4.40), rural residence (AOR:7.16;95% CI: 3.31–15.46), duration of diabetes treatment 5–10 years (AOR: 0.03; 95% CI: 0.1–0.11), duration of diabetes treatment ≥ 10 years (AOR:0.8; 95% CI: 0.03–0.21), haven’t social support (AOR: 0.10; 95% CI: 0.05–0.23), haven’t got health education (AOR: 0.17,95%CI 0.09–0.32) were factors significantly associated with self-care practice.

**Conclusions:**

Despite, the importance of diabetes self-care practice for the management of diabetes and preventing its complications, a high number of diabetes patients had poor self-care practices. Female, rural residence, duration of diabetes mellitus, lack of social support, and not get of health education were significantly associated with poor self-care practice. Therefore, health care providers should give attention to diabetic patients with the aforementioned factors that affect diabetic patients’ self-care practices.

## Introduction

Diabetes mellitus (DM) is a chronic disease that affects both developed and developing countries, requiring lifelong treatment and patient participation [1]. DM is one of the four non-communicable diseases that are growing to pandemic proportions [2,3]. Despite significant progress in diabetes management in recent years [4], diabetes remains one of the primary causes of morbidity and mortality in the world [5,6].

Globally, DM affects 463 million individuals (9.3 percent) and probably impact 537 million people by 2021. The total population with diabetes is predicted to reach 643 million by 2030 and 783 million by 2045. Diabetes-related disasters 6.7 million fatalities and at least 966 billion USD in medical costs [7]. Ethiopia has 800,000 diabetes patients in 2000, and the figure is predicted to rise to 1.8 million by 2030 [8].

Diabetes mellitus is a lifelong disease that needs behavioral modifications and effective self-care practice approaches for better glycemic control [9]. Self-care practices are activities that people with diabetes or those who are at risk of getting diabetes do their management, improvements in glucose management, fewer problems, and a higher quality of life are all linked to self-care behaviors [4,10]. Without appropriate self-care practice, targeted therapeutic management is challenging or has negative effects if not addressed promptly [9,11].

To reduce the strain on existing health systems and those affected diabetic patients must engage in particular diabetes self-care practices. It is an important way of developing diabetes knowledge and practice to cope with the complicated nature of diabetes in a social setting [5,12]. Raised blood pressure, tobacco use, alcohol intake, physical inactivity, poor eating choices, and being overweight are risk factors for diabetes [13], poor medication adherence and poor self-care habits have also been identified as a barrier to effective diabetes management [13,14].

The American Association of Diabetes Educators (AADE) model recommends that self-care practices include a continuous set of activities like healthy coping, healthy eating, being active, taking medications, self-monitoring blood glucose, risk reduction, and problem-solving for the best health outcomes and quality of life [15]. Diabetic self-care practice is the act of initiating and carrying out actions them live a healthy [16], a person’s habit reflects agreed-upon instructions from a healthcare professional, such as following a prescribed diet, routine foot care, self-monitoring of plasma glucose, and/or other lifestyle modifications [17].

In Sub-Saharan Africa, like Ethiopia diabetes self-care practice was poor, affectation a serious threat to the health system of the population [18]. However, in a developing country like Ethiopia, where resources are limited and treatment costs are rising, focusing on self-care management may result in improved treatment outcomes and lower drug-related costs. To the best of our knowledge, there is a limited study on diabetes self-care practice in the study area. As a result, the focus of these investigations is on the extent of self-care practice and associated factors among diabetic patients in the Gurage Zone, southwest Ethiopia.

## Methods and materials

### Study design, area, and period

An institution-based cross-sectional study was conducted at public hospitals in Guraghe Zone. Gurage zone is located 158 km south of Addis Ababa capital city of Ethiopia. Zone has Seven Hospitals to serve chronic disease, with separate diabetes, hypertension, and heart failure follow-up clinic. For people living with diabetes that were on follow-up in the out-patient department of the diabetic clinic, treatment has been provided two days a week, during the study period 630 DM patients per month attend hospitals. Based on the 2007 Ethiopian national population and housing census, the population of the Gurage Zone is projected to be about 1,609,908, and 51.38% are females. The study period is from February 6^th^ to March 29^th,^ 2021.

### Source and study population

The source population consisted of all diabetic patients who attend diabetics’ clinics at the Gurage zone hospital, while the study population consisted of all diabetic patients who had follow-up during the data collection period.

### Inclusion and exclusion criteria

The study included all diabetic patients whose ages were>18 years old at the public hospital of Gurage zone who had received at least six months of treatment. Participants who were seriously ill had gestational diabetes, and were unable to hear or speak were excluded from the study.

### Sample size determination and sampling procedures

Using a single population proportion formula, the sample size was calculated. With the assumption of a 5% margin of error, a 95% confidence interval (CI), and a 5% non-response rate, the percentage of DM self-care practice from the prior study in the Tegray region (46.7%) [19].

Thus the sample size was

$$n=\frac{\left(Z\;\alpha /2)2\;P(1-P\right)}{d2}$$

$$n=\frac{\left(1.96)2\;0.467(1-0.467\right)}{(0.05)2}=383$$

The total number of samples by adding 10% non-response was 420. A systematic random sampling technique was employed to select the study participants. A total of 630 diabetes patients were registered per month for diabetes follow up then we calculated the K^th^ interval that was 3. So, we interview the study participants every 3 diabetes patients. The diabetic unit’s documented diabetic patients were employed as a randomization sampling strategy. Study subjects were selected every three intervals until to reach the total sample size and the first participants were selected by lottery method.

### Data collection and procedure

Data were gathered using a standardized questionnaire that was administered by an interviewer diabetic self-care practices in diabetic patients who had medical checks at the Gurage zone hospital’s diabetes clinic. The WHO resources and other similar studies were used to develop the data collection tool. We use six BSc nurses were the data collectors, and senior supervisors were closely monitoring the data collection process at each site. Details about self-care practice were collected using the summary diabetes self-care activities (SDSCA) [20].

The questionnaire has four parts: socio-demographic characteristics, the second part measures knowledge-related factors, the third part measures self-care behaviors, and the fourth section measures your diabetic self-care activities during the last seven days.

According to WHO criteria, body mass index (BMI) was classified as underweight if it was less than 18.5, normal weight if it was 18.5–24.9, overweight if it was 25–29.9 kg/m2, and obese if it was greater than 30 kg/m2 [21]. The revised version of the SDSCA questionnaire, taken from the previous study originally developed by Schmitt et al., was used to measure participants’ self-reported frequency of adhering to self-care practice [22].

### Data quality control

Data quality was ensured by carefully designing the data collection technology, making relevant modifications, and recruiting the right participants. Both data collectors and supervisors received one-day training. Data were cleaned, coded, and double-checked for accuracy and consistency. The structured questionnaire was written in English first, and then translated into the local language (Amharic), and then back into English to ensure consistency. A Cronbach alpha score of 0.82 was used to assess the tool’s internal dependability. Every day the collected data were reviewed and checked for completeness and consistency of the response.

### Study variables

#### Dependent variable

Diabetes self-care practice.

#### Independent variable

**Socio-demographic** factors age, sex, residence, educational status, occupation, marital status, religion, and estimated monthly income of the respondents.

**Clinical characteristics**: duration of DM, social support, Comorbidity and treatment modalities Body mass index (BMI) and glycemic control level.

**Knowledge** status of the participants: about exercise, diet, smoking, and drinking.

**Behavioral variables**: History of smoking and history of alcohol, Consumption has been assessed during a lifetime and exercise.

### Operational definitions

**Self-care practice:** The SDSCA diabetes self-care practice scale was used to measure participants’ diabetic self-care practices, which included food, physical activity > 30 min/day, self-blood glucose testing, and foot care practices in the seven days before the study [23]. Detail about self-care activities was collected using SDSCA [20].

**Good self-care practice**: These four domains were used to assess participants, and the frequency of each self-care practice was recorded. The above mean from the total self-care practice measurement items was considered a good self-care practice [24].

**Poor self-care practice**: These four domains were used to assess participants, and the frequency of each self-care practice was recorded. The less than mean from the total self-care practice measurement items was considered a good self-care practice [20].

**The level of glycemic control** was indicated as ‘adequacy glycemic control ‘when fasting blood sugar (FBS) results were less than 126mg/dl(7mm) i.e. an average of three visits) or when RBS results were less than 200/dl, ‘**inadequate glycemic control’** takes place when a parameter is beyond the criteria of adequate glycemic control [25].

**Physical activity**: at least 30 minutes of aerobic activity per day or ≥ 3 days per week [26].

### Data processing and analysis

The collected data were double-checked for their completeness. The data was then entered into Epi-data version 3.1 and exported to SPSS version 23, where it was cleaned, modified, and coded. Binary logistic regression was run to see the crude significant relations of each independent variable with diabetes self-care practice. In the bi-variable logistic regression analysis, a p-value < 0.25 were entered into multivariable logistic regression. Finally, significant factors were identified based on 95% confidence level p-value < 0.05. The Hosmer-Lemen show test was performed to determine the model’s goodness of fit.

### Ethics approval and consent to participate

A permission letter was obtained from the Gurage zone health department and the associated hospitals’ administrative offices, as well as ethical clearance from Wolkite University Ethical Review Board (ERB). The responders gave their written informed consent in writing. All required precautions were taken to ensure the confidentiality of the patients’ information and all of their advantages. Respondents are free to leave the interview at any time.

## Results

### Socio-demographic characteristics of participants

Out of the total 420 eligible participants, 384 of them had completed the study questionnaires, which gave a response rate of 91.4%. Of the total of respondents, 254 (66.1%) were male, 290 (75.5%) were married, 77 (20.1%) were single, and 17 (4.4%) were divorced. The mean ±SD age of participants was (43.54 ±12.38) years, 316 (82.3%) were rural residents, and only 17 (4.4%) were a member of the Ethiopian diabetic association.

Regarding their level of education, 118 (30.7%), 113 (29.4%), and 34 (8.9%) of the study’s participants were, respectively, illiterate, can read and write, and college and above. The majority 305 (79.4%) of study participants’ monthly incomes were less than USD 97.77 (Table 1).

**Table 1:** Socio-demographic characteristics of patients with diabetes mellitus attending diabetic clinic follow-up Gurage zone public Hospital south Ethiopia 2021 (n = 384).

Variables	Category	Frequency	Percentage
Sex	female	254	66.1
	Male	130	33.9
Age in years	18–40	189	49.2
	41–59	152	39.6
	>60	43	11.2
Marital status	Single	77	20.1
	Married	290	75.5
	Divorce	17	4.4
Educational status	Illiterate	118	30.7
	Can read &write	113	29.4
	Primary school	37	9.6
	Secondary school	82	21.4
	College and above	34	8.9
Occupation	Farmer	214	55.7
	Government employee	65	16.9
	Merchant	45	11.7
	Self-employee	60	15.6
Residence	Rural	316	82.3
	Urban	68	17.7
Member of Ethiopian diabetic association	Yes	17	4.4
	No	325	84.6
	Don’t know	42	10.9
Monthly income	97.77USD	305	79.4
	97.77USD	79	20.6

### Clinical characteristics of the study participants

The mean duration of medical diagnosis for diabetes mellitus was 2.00 (±0.52) years and the majority 309 (80.5%) of the study participants have no social support. The majority 292 (76%) of the participants had drunk alcohol, and 97 (25.3%) of the study participants had Comorbid disease. only 25 (6.5%) participants had a family history of DM. Regarding complications, 34 (8.9%) of participants had developed complications. More than half 207 (53.9%) of the participants were normal BMI, and 158 (41.1%) of the participants were overweight (Table 2).

**Table 2:** Clinical characteristics of the adult diabetic patient in Gurage Zone Hospital 2021(n = 384).

Variables	Category	Frequency	Percentage
Family history of diabetes	Yes	25	6.5
	No	359	93.5
Duration of living with diabetes in years	<5	51	13.3
	5–10	282	73.4
	>10	51	13.3
Other chronic illness	Yes	97	25.3
	No	287	74.7
Cigarette smoke	Yes	51	13.3
	No	333	86.7
Social support	yes	75	19.5
	No	309	80.5
Drink alcohol	Yes	292	76
	No	92	24
Type of anti-diabetic drugs	Oral	270	70.3
	Insulin	114	29.7
Regular exercise	Yes	163	42.4
	No	221	57.6
BMI (kg/m^2^)	Underweight	3	.8
	Normal weight	207	53.9
	Overweight	158	41.1
	Obese	14	3.6
Having Glucometer	Yes	55	13.3
	No	333	86.7
Develop diabetic complication	Yes	34	8.9
	No	350	91.1
Get health education	Yes	238	62
	No	146	38
Overall self-care practice	Good	152	39.6
	Poor	232	60.4

### Distribution of self-care practice status among diabetic patients

In the Gurage Zone hospital’s diabetic clinic, among 384 participants in the study, more than half them 232 (60.4%) had poor self-care practices 145 (37.8%) males participants had poor self-care practices, and 87 (22.7%) female participants had good self-care practices (Table 2).

### Factors associated with diabetic self-care practice

After the binary logistic regression, variables with a P-value of less than 0.25 were considered for the multivariable logistic regression. Regarding bivariate analysis, sex, occupation, place of residence, duration of, having social support, and having health education were significantly associated with diabetic self-care practice.

These factors were used in the multivariable regression analysis, which revealed, being females were 2.4 times more likely to have poor self-care practice than males (AOR; 2.40, 95% CI 1.31–4.40), and those participants who lived in the rural areas were 7 times more likely to have poor diabetic self-care than their counterpart (AOR; 7.16; 95% CI 3.31–15.46). Duration of diabetes 5–10 years was 97% less likely to perform poor self-care practice compare to < 5 years (AOR; 0.03; 95% CI 0.1–0.11), duration of diabetes >10 years were 92% less likely to perform self-care practice than that duration < 5 years (AOR; 0.08; 95% CI 0.03–0.21).

Regarding having social support for diabetic patients, those who had no social support were 90% less likely to perform self-care practice than those who had social support (AOR; 0.10; 95% CI 0.05–0.23). Patients who did not get health education about diabetes were 83% less likely to engage in self-care practices than their counterparts (AOR; 0.17; 95% CI 0.09–0.32) (Table 3).

**Table 3:** Factors influencing diabetes patients’ self-care practice in Guraghe Zone Hospital southwest, Ethiopia 2021(n = 384).

Variable	Category	DM Self-care practice status		COR(95% CI)	AOR(95% CI)
		Poor n%	Good n%		
Sex	female	87(22.7)	43(11.2)	0.66(.42–1.02)*	2.40(1.31–4.40)**
	Male	145(37.8)	109(28.4)	1.00	1.00
Occupation	Farmer	123(32)	91(23.7)	2.04(1.08–3.83)*	1.09(0.36–3.30)
	G. employee	31(8.1)	34(8.9)	3.02(1.42–6.39)*	.39(0.09–1.76)
	Merchant	34(8.9)	11(2.9)	.89(.37–2.16)	.55(0.14–2.11)
	Self-employee	44(11.5)	16(4.2)	1.00	1.00
Place of residence	Rural	181(47.1)	135(35.2)	2.24(1.24–4.05)*	7.16(3.31–15.46)**
	Urban	51(13.3)	17(4.4)	1.00	1.00
Duration of living with DM in years	<5	40(10.4)	11(2.9)	1.00	1.00
	5–10	175(45.6)	107(27.9)	0.14(.06-.33)*	.03(.01–0.11)**
	> 10	17(4.4)	34(8.9)	0.31(.16–57)*	.08(.03–0.21)**
Social support	Yes	61(15.9)	14(3.6)	0.28(.15-.53)*	0.10(.05–0.23)
	No	171(44.5)	138(35.9)	1.00	1.00
DM Health education	yes	158(41.1)	80(20.8)	1.92(1.26–2.93)*	0.17(0.09–0.32)**
	No	74(19.3)	72(18.8)	1.00	1.00

*variable having p value < 0.25 in bi-variable,

**Statistical significance P value < 0.05.

## Discussion

This study aimed to assess the prevalence of diabetic patients’ self-care practice in a public hospital in the Gurage zone in southwest Ethiopia. This study revealed that 60.4% of study participants had poor self-care practices. This finding was in line with the study conducted in central Tigray hospital 62.7% [27], and west Ethiopia at 57.3% [28]. However, this finding was higher than a study conducted at Debre Tabor University 36.9% [29], in Addis Ababa Ethiopia 52% [30], Jimma medical center 45.8% [31], in Tigray 53.3% [19]. The possible reasons could be the difference in socio-cultural variation, lifestyle, and inadequate access to glucose monitoring machines, test strips, and levels of education in the patients were differences in self-care practice.

In this study, self-care practice was lower than in the previous studies conducted in Ayder Comprehensive Specialized Hospital 74.5% [32], Nigeria 67.4% [33], and Saudi 90.0% [34]. The possible reasons could be due to differences in a study area, which means the current study was conducted 82.3% in rural and semi-urban areas but the previous study was mostly in urban. Where knowledge and information on self-care practice are relatively lower compared to the previous studies conducted predominantly in urban areas. In addition to this, cultural and socio-economic features of the society were varied across the community in Ethiopia, and differences in the lifestyle of the participants.

In the current study, female diabetes patients were significantly associated with poor self-care practice when compared to their counterparts. This finding was consistent with the study conducted in Bangladesh, Gujarat, Pakistan [35–37] respectively. This could be a result of women being allocated to perform more everyday housework and having poorer self-care practice than men.

Patients who were residents of rural areas had higher odds of having poor self-care practice compared with patients who were residents of urban areas. This was supported by different studies in Karnataka [38]. The possible reasons might be good access to a balanced diet in urban, and their working condition. The reality is that the majority of the fruit and vegetable produce is grown in rural communities but because of the paucity of vegetables and fruits in the study area, even if the majority of products were sold to buy less expensive products for their family.

Respondents who had a short duration of diabetes treatment were significantly associated with poor self-care practice than with a long duration of treatment. This study is in line with studies conducted in Aresi, and Ethiopia [39,40] respectively. The probable cause might be because of regular interactions with healthcare professionals, who may have learned crucial knowledge to advance self-care practices.

Participants who had no social support were significantly associated with poor self-care practice than those who had strong social support. This study was supported by different studies in Benshangul Gumez, and a systematic study in Ethiopia [40,41] respectively. The possible cause could be social assistance from a relative in the form of financial, emotional, or informational support relating to this help to enhance the self-care practices of diabetic patients.

Furthermore, patients who have got diabetes education as preventive effect on diabetic complications and this finding was supported by a study conducted at Bahir dar, and Dilla South Ethiopia [42,43]. This might be because education inspires the individual’s performance of diabetes self-care to increase targets including blood glucose monitoring, diet maintenance, physical activity, and medical attention among adults.

### Limitation

The data collector was a health care provider which may result in socially desirable bias on self-care practice. On the other hand, the study was cross-sectional that cannot determine a cause-effect relationship.

## Conclusion

The results of our study showed that self-care practice was not widely practiced. Poor self-care practices were substantially correlated with sex, location, diabetes duration, social support, and health education. Therefore, healthcare professionals should focus on patients who have the aforementioned factors. Additionally, plans should be made to help diabetic patients’ better self-care practice.

## Supporting information

S1 File (SAV)

## Abbreviations

AADE: American Association of Diabetes Educators
BMI: Body mass index
DM: diabetes Millets
FBS: fast blood sugar
NCD: none communicable disease
RBS: random blood sugar
SDSCA: summary diabetes self-care activities
WHO: world health organization
